# Repurposing phytochemicals as anti‐virulent agents to attenuate quorum sensing‐regulated virulence factors and biofilm formation in *Pseudomonas aeruginosa*

**DOI:** 10.1111/1751-7915.13981

**Published:** 2021-11-29

**Authors:** Jatin Chadha, Kusum Harjai, Sanjay Chhibber

**Affiliations:** ^1^ Department of Microbiology Panjab University Chandigarh India

## Abstract

Unregulated consumption and overexploitation of antibiotics have paved the way for emergence of antibiotic‐resistant strains and ‘superbugs’. *Pseudomonas aeruginosa* is among the opportunistic nosocomial pathogens causing devastating infections in clinical set‐ups globally. Its artillery equipped with diversified virulence elements, extensive antibiotic resistance and biofilms has made it a ‘hard‐to‐treat’ pathogen. The pathogenicity of *P. aeruginosa* is modulated by an intricate cell density‐dependent mechanism called quorum sensing (QS). The virulence artillery of *P. aeruginosa* is firmly controlled by QS genes, and their expression drives the aggressiveness of the infection. Attempts to identify and develop novel antimicrobials have seen a sharp rise in the past decade. Among different proposed mechanisms, a novel *anti‐virulence approach* to target pseudomonal infections by virtue of anti‐QS and anti‐biofilm drugs appears to occupy the centre stage. In this respect, bioactive phytochemicals have gained prominence among the scientific community owing to their significant quorum quenching (QQ) properties. Recent studies have shed light on the QQ activities of various phytochemicals and other drugs in perturbing the QS‐dependent virulence in *P. aeruginosa*. This review highlights the recent evidences that reinforce the application of plant bioactives for combating pseudomonal infections, their advantages and shortcomings in anti‐virulence therapy.

## Graphical abstract

The review sheds light on the colossal literature that provides us a detailed insight into the invincible quorum sensing (QS) mechanisms in the versatile human pathogen, *Pseudomonas aeruginosa*, its intricate association with the regulation of virulence factors and biofilm formation, and how bioactive phytochemicals can be exploited disarm this pathogen.

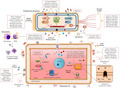

## Introduction

Perpetual consumption of antibiotics for antimicrobial therapy has led to the widespread emergence of antimicrobial‐resistant bacterial strains worldwide (Chadha and Khullar, 2021). Today, antimicrobial resistance (AMR) is one of the gravest public health issues. Pathogens have evolved into multi‐drug‐resistant (MDR) varieties as a consequence of overexploitation and unregulated usage of antibiotics (Chadha, 2021, 2021). Confining to this, the ESKAPE pathogens, *Enterococcus faecium*, *Staphylococcus aureus*, *Klebsiella pneumoniae*, *Acinetobacter baumannii*, *Pseudomonas aeruginosa* and *Enterobacter* sp., have posed a severe challenge in combating infectious diseases (Mulani *et al*., 2019). The race between the discovery of newer antibiotics and superbug evolution is inclined towards the latter end. According to the statistics of the UN Ad hoc Interagency Coordinating Group on Antimicrobial Resistance, global drug‐resistant infections could inflict 10 million deaths each year by 2050 and impel 24 million people into extreme poverty by 2030 (World Health Organization, 2019). This scenario is grimly reflected in developing countries such as India, where the antibiotic consumption rates have nearly doubled (Taneja and Sharma, 2019). Alarmingly, one‐fifth of the world’s AMR‐associated deaths have been projected to occur in India by 2050 (Dixit *et al*., 2019). Realizing this, the United Nations General Assembly at New York (2016) conducted a high‐level meeting to address the global AMR issue (Laxminarayan *et al*., 2016). Therefore, it is a global need to armour alternative intervention and therapeutic stratagems against the rising burden of AMR. In this direction, secondary plant metabolites and bioactive phytochemicals have regained immense prominence among the scientific community. The roots of herbal medicine originate from the Ayurveda, Siddha and Unani, the ancient healthcare systems of Indian medicine derived from the Indian ancient literature: ‘Vedas’ (Sen and Chakraborty, 2017). Research on traditional medicines has proven to be a cynosure for pharmaceutical industries as plants harbour repertoires rich in secondary metabolites such as alkaloids, flavonoids, terpenoids, tannins, saponins and polyphenols, which have been shown to resist pathogens and simultaneously boost the immune system (Behl *et al*., 2021). Their antimicrobial potential has proved to be beyond comprehension, with promising efficacy at extremely low doses (Chadha *et al*., 2021c). The antimicrobial properties of plants have been favoured worldwide due to their natural existence, easy and economic availability, formidable pharmacological properties and negligible cytotoxicity with the least probability of developing resistance compared to antibiotics (Cheesman *et al*., 2017). Despite the advent of molecular docking, drug discovery development and the rise of the ‘omics era’, bioactive phytochemicals prove to hold strong and fertile ground for their therapeutic application in medicine.

Among the ESKAPE pathogens, *Pseudomonas aeruginosa* is an extensively versatile gram‐negative opportunistic pathogen associated with nosocomial infections (Kerr and Snelling, 2009). It is highly invasive and toxigenic, and possesses an arsenal of diverse virulence factors ranging from the secretion of extracellular enzymes to exotoxins and biofilms to alluring but disarming pigments (Chadha *et al*., 2021a). Nevertheless, its ability to be ubiquitous, furnished with remarkable tissue tropism and a multitude of intrinsic and acquired antimicrobial resistance mechanisms make it an obdurate and notorious pathogen (Pang *et al*., 2019). These factors synergistically orchestrate the manoeuvring of the host immune system and destruction of host tissues leading to high morbidity and mortality in immunocompromised individuals. *P. aeruginosa* is well known to be associated with respiratory tract infections, particularly pneumonia and chronic bronchitis in patients suffering from cystic fibrosis, acquired immune deficiency syndrome (AIDS), severe burn wounds, and gastrointestinal, osteomyelitis, bone and joint infections (Bodey *et al*., 1983). A major proportion of pseudomonal manifestations result from urinary tract infections inflicted due to the insertion of medical devices such as urinary catheters (Cole *et al*., 2014). Its role in the development of devastating eye infections such as keratitis, conjunctivitis, endophthalmitis, dacryocystitis and corneal ulcers with permanent loss of vision is well documented (Ferreiro *et al*., 2017). Apart from this, *P. aeruginosa* is also linked with septic shock, bacteraemia, meningitis, endocarditis and brain abscess in patients undergoing neurological procedures (Shah *et al*., 1999).

**Fig. 1:**
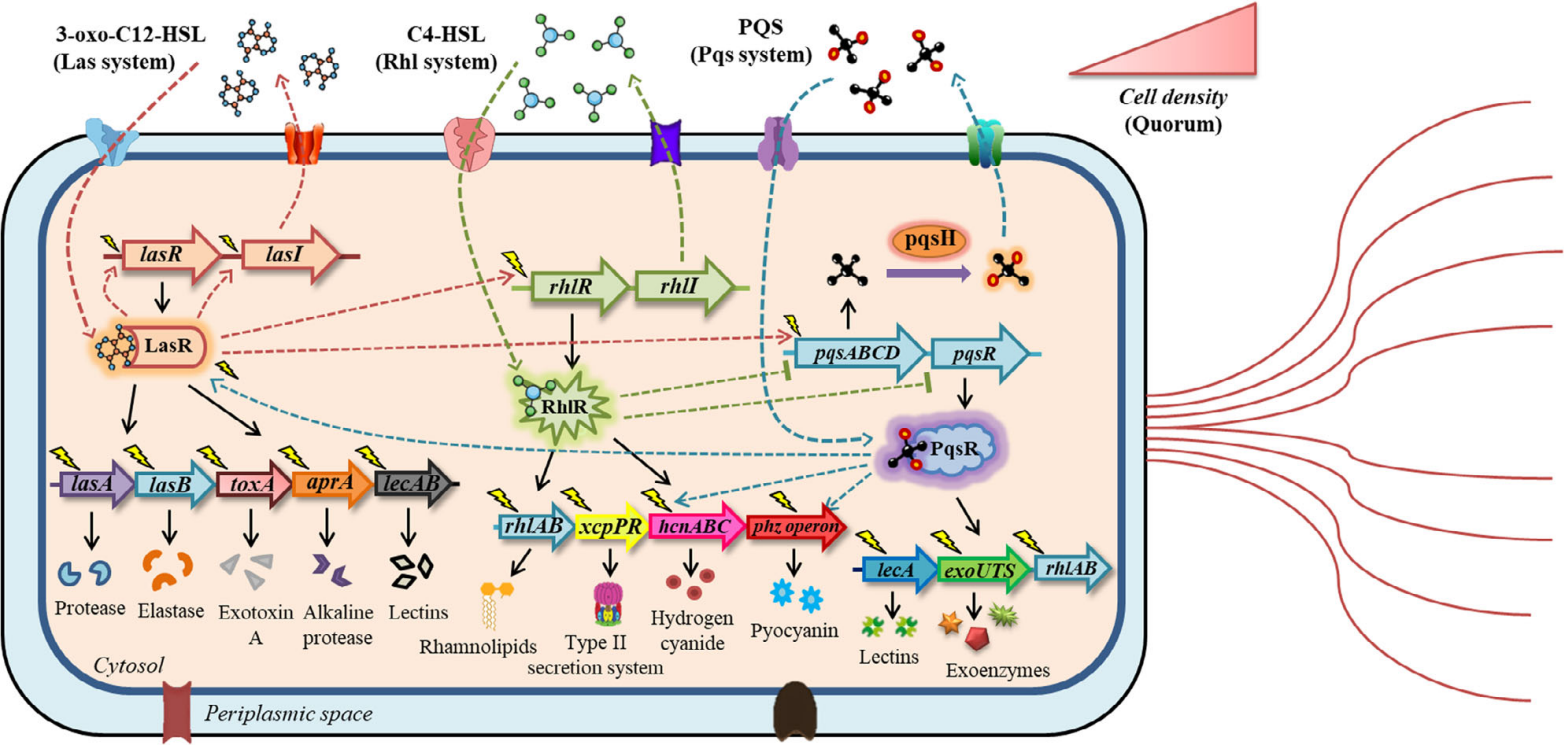
QS circuitry of *P. aeruginosa*. The complex intracellular signalling is regulated by Las, Rhl and Pqs systems using their cognate QSSMs: 3‐oxo‐C12‐HSL, C4‐HSL and PQS respectively. Induction of these QS systems enhances the virulence of *P. aeruginosa*, driving disease progression and worsening the severity of infection. The figure has been made based on existing literature cited in this review using CorelDRAW Graphics Suite 12.

The pathogenicity of *P. aeruginosa* is regulated by a multitude of concatenated mechanisms, which in turn are stringently governed by a phenomenon called quorum sensing (QS) (Lee and Zhang, 2015). This resilient pathogen communicates and harmonizes at the intercellular level by releasing diffusible signalling molecules called autoinducers (AIs). The intricacy of the QS system in *P. aeruginosa* lies in the fact that the synthesis of AIs depends on the pathogen's population density, which in turn corresponds to the expression of virulence factors, biofilm formation, progression and severity of the disease (Erickson *et al*., 2002). We have augmented the findings based on pre‐existing and recent literature and illustrated the QS circuitry of *P. aeruginosa* in Fig. 1 along with the virulence machinery each QS system activates, driving the progression of bacterial infection. This magnificent bacterial communication system has drawn tremendous attention from microbiologists because of its involvement in acute and chronic human infections. In this context, the global effects of the three QS systems in regulating the QS‐associated virulence hallmarks of *P. aeruginosa* have been described recently (Chadha *et al*., 2021a). Thus, targeting the QS system can be a possible strategy to combat pseudomonal infections since it would directly impact the downstream virulence factors. This includes the QS inhibitors (QSIs) that target signal output, accelerating the degradation of AIs, or inhibit signal reception by directly associating with AIs, thereby armouring anti‐QS response called quorum quenching (QQ). Since QS contributes like an auxiliary factor towards regulating virulence elements in *P. aeruginosa*, its abrogation using QSIs does not exert any selection pressure for the emergence of any anthropogenic resistance, which is generally seen upon conventional antibiotic administration (Rasmussen and Givskov, 2006). Additionally, a fascinating aspect of QSIs is that they attenuate the QS circuits of a particular pathogen at sublethal concentrations (sub‐MICs) without posing any stress or survival threats (Kalia *et al*., 2019). Hence, this seems to be a promising anti‐virulence therapy to tackle pseudomonal infections. A plethora of traditional medicinal plants, herbs and their constituent phytochemicals that have been elucidated to exhibit prolific QQ potential against *P*. *aeruginosa* is also considered safe for therapeutic use (Vandeputte *et al*., 2010; Kumar *et al*., 2015; Lou *et al*., 2019; Topa *et al*., 2020). QSIs derived from various natural and synthetic origins have been documented to attenuate the virulence machinery in *P. aeruginosa* (Kalia, 2013). Their deployment in the biotechnological arena to counter human infections has recently been described (Kalia *et al*., 2019). Considering the colossal literature and tremendous potential of phytochemicals in therapeutics, this review is a humble attempt to recapitulate the findings by implicating the anti‐virulent properties of plant bioactives in attenuating QS and virulence in *P. aeruginosa*.

## Quorum Sensing circuitry: the dictator for pseudomonal virulence

Intercellular communication in bacteria stimulates coordinated responses to display a multitude of virulence traits that are not possible to achieve for individual cells. The signalling network in *P. aeruginosa* is perhaps one of the most complex yet profoundly studied examples among all the QS systems. It consists of multiple concatenated signalling pathways that synergistically regulate bacterial virulence, making *P. aeruginosa* a highly notorious and deadly human pathogen (El Zowalaty *et al*., 2015). The QS circuitry in *P. aeruginosa* represents a global regulatory system that directly or indirectly controls the expression of more than 10% of its genome (Schuster and Greenberg, 2006) and about 20% of the bacterial proteome (Deep *et al*., 2011). QS in *P. aeruginosa* is mediated through three main systems, termed Las, Rhl and Pqs. The LasR/LasI and RhlR/RhlI QS systems regulate the synthesis and signal transduction *via N‐*3‐oxo‐dodecanoyl‐L‐homoserine lactone (3‐oxo‐C12‐HSL) and *N‐*butyryl‐L‐homoserine lactone (C4‐HSL) AIs respectively. LasI synthesizes 3‐oxo‐C12‐HSL, which activates the cytoplasmic receptor, LasR, which regulates the expression of genes associated with biofilm formation, haemolysins, proteases, elastases and exotoxin A production (Rutherford and Bassler, 2012). Similarly, RhlI synthesizes the C4‐HSL, which associates with its cognate receptor RhlR and drives the expression of virulence genes responsible for producing pyocyanin, hydrogen cyanide, siderophores, elastases and alkaline protease, and regulating bacterial motility (Papenfort and Bassler, 2016), while the Pqs system is modulated using non‐AHL signalling molecules called alkyl‐4‐quinolones. PQS, structurally known as 2‐heptyl‐3‐hydroxy‐4‐quinolone, is chemically isolated from the AHL‐mediated Las and Rhl systems (Reyes *et al*., 2020). It modulates the expression of genes involved in biofilm formation, inducing swimming motility and production of pyocyanin, proteases, elastases, rhamnolipids, pyochelin and pyoverdine siderophores, which favour immune evasion and induce lysis of neutrophils, macrophages and other host cells. Pqs null mutants have been demonstrated to exhibit avirulent phenotype with inhibition of biofilm development and production of elastase, pyocyanin, lectins and rhamnolipids (Jander *et al*., 2000; Cao *et al*., 2001; Diggle *et al*., 2003). Although most of the virulence determinants of *P. aeruginosa* are regulated through QS, others such as type III secretion system (T3SS) and lipopolysaccharide (LPS) production are not (De Kievit *et al*., 2001; Pena *et al*., 2019). Apart from these three QS systems, the last decade witnessed speculations over the involvement of a fourth QS system in *P. aeruginosa*, called the integrated quorum sensing (IQS) pathway. However, studies have recently discredited its role in QS and asserted that IQS is an aeruginaldehyde and a by‐product of pyochelin biosynthesis or degradation, putting an end to the previous conceptions of IQS to be a product of *ambBCDE* genes (Cornelis, 2020). Nevertheless, these three QS circuits play a quintessential role in the fortification and augmentation of virulence factors in *P. aeruginosa*.

Over the years, scientists have proposed different models describing the cross‐talk between different branches of the QS signalling network. These have been in the limelight and sparked critical debates over the interaction of various QS systems in *P. aeruginosa*. In this regard, Lee and Zhang (2015) proposed that the three QS systems in *P. aeruginosa* are interconnected hierarchically, with the Las system occupying the keystone position regulating the signalling hierarchy. It has been shown to regulate both Rhl and Pqs systems (Lee and Zhang, 2015). Interestingly, a circular model establishing the interconnections between the QS systems in *P. aeruginosa* has been proposed recently (Maura *et al*., 2016). It was documented that MvfR (PqsR) directly controls the expression of *lasR* and *rhlR* during early phases of bacterial growth with RhlR acting as a direct repressor of the MvfR (PqsR) QS system during late exponential and stationary phases (Maura *et al*., 2016). On the contrary, binding of MvfR to *lasR* induces *mvfR* expression and the PqsH‐assisted conversion of HHQ into PQS, thereby increasing MvfR activity. These mechanisms were shown to function as stringent autoregulatory loops, dictating the QS circuitry in *P. aeruginosa*. Nevertheless, the QS systems of *P. aeruginosa* communicate with one another *via* chemical molecules known as quorum sensing signalling molecules (QSSMs). The LasR‐3‐oxo‐C12‐HSL complex multimerizes and activates the transcription of *rhlR*, *rhlI* and *lasI*, and other pseudomonal virulence genes, resulting in a positive feedback loop (Kiratisin *et al*., 2002). The RhlR‐C4‐HSL complex also dimerizes and activates the expression of RhlI and its own regulon, thereby generating a second positive feedback loop (Ventre *et al*., 2003). LasR‐3‐oxo‐C12‐HSL complex regulates PqsR, the transcriptional regulator of *pqsABCD* operon stimulating the biosynthesis of PQS and the transcription of *pqsH*, the gene encoding a probable monooxygenase (Schertzer *et al*., 2010). Additionally, PQS has been found to enhance the transcription of *rhlI*, thereby influencing the C4‐HSL output and cumulative impact of the Rhl system (McKnight *et al*., 2000). Also, the expression of *pqsR* and *pqs* operon is inhibited by RhlR‐C4‐HSL, suggesting that the concentration ratio between 3‐oxo‐C12‐HSL and C4‐HSL plays a decisive role in the dominance of the Pqs signalling system (Cao *et al*., 2001). Although the Las and Pqs systems regulate the Rhl pathway like a QS‐command workhorse, most virulence factors dependent on QS are principally triggered by the RhlR‐C4‐HSL complex. Therefore, these systems represent a cascade mechanism commanding the virulence artillery of *P. aeruginosa* after achieving a quorum. This magnificent bacterial communication system has drawn tremendous attention from microbiologists because of its involvement in acute and chronic human infections. Deciphering the precise molecular mechanisms and potential targets behind these communication systems can help scientists in developing novel antimicrobial drugs that will help in targeted and combative therapy.

## Orchestrating bioactive phytochemicals as potential therapeutics against *P. aeruginosa*: evidences from studies

**Fig. 2:**
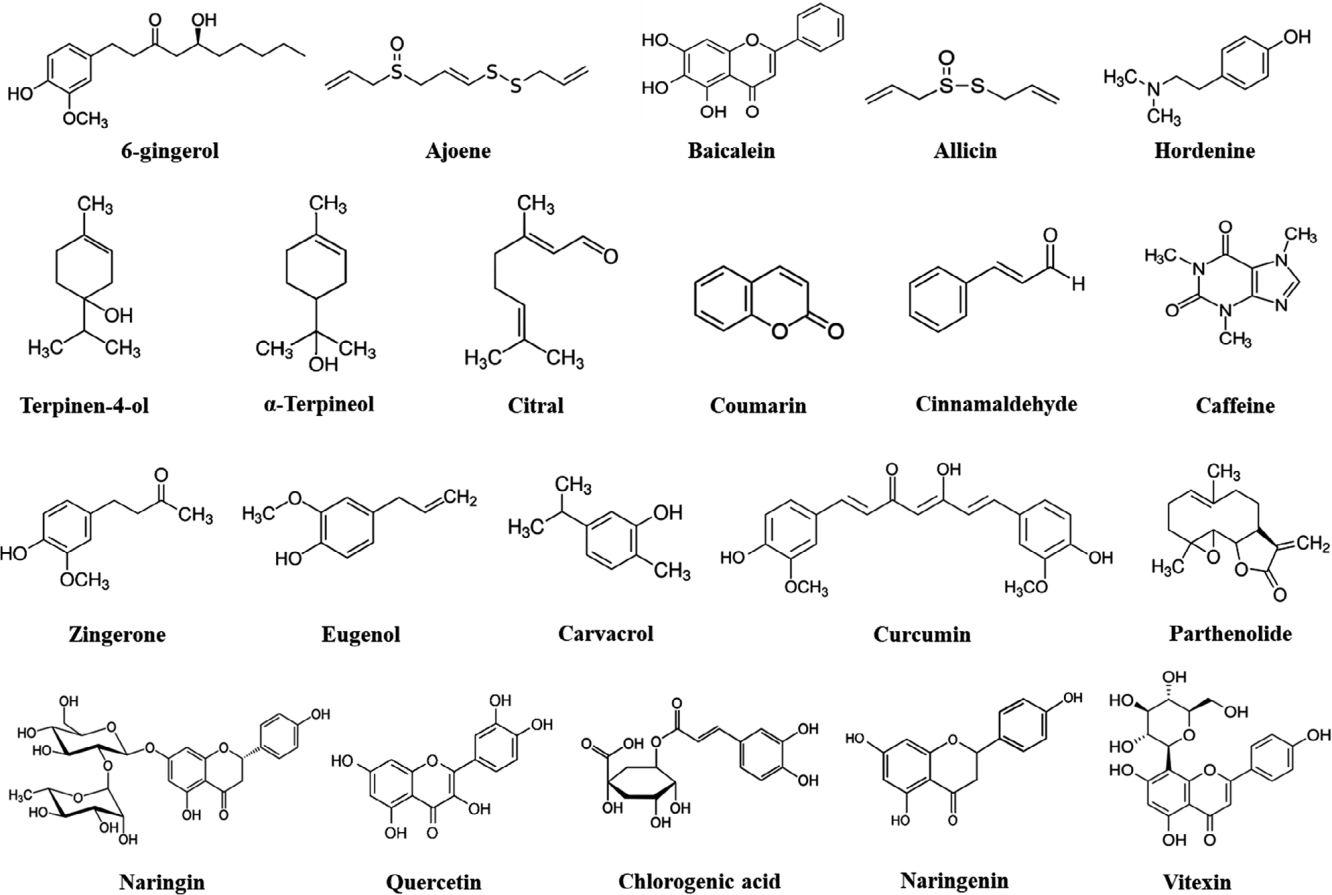
Chemical structure of plant bioactives reported to exhibit quorum quenching and anti‐virulence properties against *P. aeruginosa*. Structure of phytochemicals cited in this review has been drawn using ChemDraw Professional 17.0 (Perkin‐Elmer, Shelton, CT, USA).

With the rise of advanced molecular techniques, countless researches across the globe are attempting to unravel the potential of novel antimicrobial. Unprecedented explorations during the 21^st^ century have successfully established the mantle of bioactive phytochemicals in combating *P. aeruginosa* by impeding its QS, thereby attenuating its virulence factors and abrogating biofilm formation. Virulence factors and QS may be targeted by plant bioactives at distinct levels and/or multiple steps. Diverse plant bioactives and their derivatives have also been documented to attenuate the swimming, swarming and twitching motilities, which play a pivotal role in bacterial adhesion and biofilm formation in *P. aeruginosa* (Khan *et al*., 2019). We have depicted the chemical structures of bioactive phytochemicals reported to exhibit QQ, anti‐virulence and anti‐biofilm activities against *P. aeruginosa* in Fig. 2. It is evident from the figure that most QQ phytochemicals share a heterocyclic ring structure similar to that of AHL molecules. This structural confirmation may permit stable interactions with the QS receptors and their ability to degrade signal receptors (Kalia, 2013). For instance, ajoene, allicin and curcumin have structures similar to AHL side‐chains but with different oxygenation levels, while in 6‐gingerol, zingerone, eugenol, carvacrol and cinnamaldehyde, the lactone ring is replaced by an aromatic moiety making it challenging to open up. A similar trend can be seen with naringin, quercetin, naringenin, vitexin and baicalein, which share a complex polycyclic structure. Moreover, *in silico* studies have revealed that these phytochemicals strongly associate with the different QS receptors of *P. aeruginosa* (Kumar *et al*., 2015; Rathinam *et al*., 2017; Quecan *et al*., 2019; Bose *et al*., 2020a). However, these have not been confirmed by any interaction studies due to the non‐proteinaceous nature of bioactive phytochemicals. Nevertheless, findings from molecular docking experiments have been positively correlated with the downregulation of essential QS genes in numerous studies, both *in vitro* and *in vivo* (Kumar *et al*., 2015; Xu *et al*., 2019; Bose *et al*., 2020b; Sharma *et al*., 2020b). This has repurposed plant‐based bioactives as multifaceted compounds holding no bar for their deployment as futuristic anti‐virulence therapies.

### 6‐gingerol

Ginger oil, derived from *Zingiber officinale* (ginger), contains this phenolic bioactive that activates spice receptors located on the tongue. Kim *et al*. (2015) established the anti‐quorum potential of 6‐gingerol against *P. aeruginosa* using standard assays *in vitro*. At a concentration of 100 µM, 6‐gingerol significantly downregulated the expression levels of *lasR* and *rhlR* alongside rhamnolipid production by about 60%. It was also predicted that 6‐gingerol could directly associate with the QS master regulator LasR protein *in silico*, abrogating its functional role in the production of exoproteases, pyocyanin and pseudomonal biofilm. Moreover, the transcription of PQS operons, *phnAB* and *pqsB*‐E, T3SS‐related genes, QS‐regulated ExoT and elastase was also repressed by 6‐gingerol. More recently, 6‐gingerol analogs have been employed as biofouling agents in reverse osmosis (RO) systems (Ham *et al*., 2019). Gingerol analogs disrupted QS activities and inhibited biofilm formation on RO membranes at significantly higher rates than 6‐gingerol itself (~ 37% analogs vs. 22% gingerol), without altering them chemically or physically. The binding affinity of gingerol analogs to QS protein receptor LasR was also enhanced coinciding with the repression of *lasB* gene by 78%.

### α‐Terpineol

It is a terpene alcohol and an integral component of essential oils extracted from pine, tea tree, *etc*. acclaimed for its application in the fragrance industry. Preliminary screening of plant volatiles provided the first insight into a possible role of α‐terpineol in QQ (Ahmad *et al*., 2015a). It was reported to inhibit violacein production in an established QS biosensor strain of *Chromobacterium violaceum* by more than 90%. In recent years, this hypothesis was affirmed by Bose *et al*. against *P. aeruginosa* using free and nanostructured lipid carriers (NLCs) containing formulations of α‐terpineol (Bose *et al*., 2020b). Expression levels of key QS genes (*las*, *rhl*) and virulence‐encoding genes including *rhlAB*, *aprA*, *lasB*, *toxA* and *plcH* were significantly diminished upon treatment with α‐terpineol. Swimming motility and biofilm formation were greatly reduced, as with the case of EPS, haemolysin, elastase and pyocyanin production. *In silico* analysis also suggested a strong binding affinity of α‐terpineol towards the QS receptors, LasR, RhlR and PqsR, accounting for its far‐reaching effects *in vitro*. Furthermore, attempts to investigate the therapeutic role of α‐terpineol have been undertaken *in vivo*. A follow‐up study in the murine keratitis model reported diminished bacterial count in corneal tissues with improved corneal histopathology and decreased inflammatory markers such as myeloperoxidase and reactive nitrogen intermediates upon treatment with nanolipoidal α‐terpineol (Bose *et al*., 2021). Interestingly, treatment with nanolipoidal α‐terpineol exerted an anti‐biofilm effect on *P. aeruginosa* and stimulated immunomodulatory responses by enhancing the production of inflammatory cytokines, IL‐2, TNF‐α and macrophage inhibitory protein‐2 in the infected eyes.

### Ajoene

Ajoene, an organosulfur bioactive derived from *Allium sativum* (garlic), has been extensively studied as a potent inhibitor of QS and QS‐derived virulence factors. The first study in this regard surfaced when Harjai *et al*. (2010) showed that crude extracts of garlic significantly reduced the synthesis of QS signals and virulence machinery in *P. aeruginosa in vitro*. Consequently, *P. aeruginosa*‐induced pyelonephritis mice with orally administered garlic extracts exhibited lower renal bacterial counts with their kidneys protected from any tissue destruction as compared to their untreated counterparts. However, this bioactive was identified from garlic extracts by Jakobsen and colleagues (Jakobsen *et al*., 2012). It was determined that ajoene downregulated the expression of key QS‐regulated genes: *lasA*, *chiC*, *lecA*, *rhlA*, *rhlB* and *prpL* in a concentration‐dependent manner *in vitro*. Moreover, the anti‐QS activity of ajoene was examined using reporter assays. Ajoene (100 µg ml^‐1^) attenuated biofilm production in *P. aeruginosa* by rendering them rhamnolipid deficient, thereby completely abolishing the lytic necrosis of PMNLs. It was even demonstrated in a murine model of pulmonary infection, where administration of ajoene (12.5µg g^‐1^) resulted in clearance of bacterial cells as compared to untreated mice.

Combinational anti‐virulence therapy using ajoene–ciprofloxacin against pseudomonal biofilms and biofilm‐associated murine acute pyelonephritis has been investigated (Vadekeetil *et al*., 2016). The compounds were reported to function synergistically *in vitro* by inhibiting biofilm formation and swimming, swarming and twitching motilities in *P. aeruginosa*. Also, *in vivo* efficacy of this combinational administration was proved to be prolific with significantly reduced bacterial load in the renal tissue of treated mice in comparison with that of their infected controls and solo drug treatments. Alternate molecular mechanisms underlying the QS inhibition by ajoene have also emerged. Ajoene was reported to repress QS in *P. aeruginosa* by the virtue of small regulatory RNA (sRNA) inhibition (Jakobsen *et al*., 2017). Using reporter assays and qRT‐PCR, the expression of RsmY and RsmZ sRNAs was found to be greatly reduced by ajoene in a dose‐dependent manner during the early‐stationary phase of bacterial growth. Recently, structural analogs of ajoene have also come into the limelight as potent inhibitors of QS mechanisms. These ajoene analogs were reported to successfully target QS activity, thereby inhibiting the virulence factors including the production of elastase, pyocyanin and rhamnolipids *in vitro* (Fong *et al*., 2017). The compounds were also shown to impede and weaken biofilm formation on implants by *P. aeruginosa* in a murine infection implant model.

### Allicin

Garlic is a rich source of antioxidants, and anti‐inflammatory and antimicrobial compounds. Another principle bioactive phytochemical derived from garlic is allicin (diallylthiosulfinate), an organosulfur compound also called as the ‘heart of garlic’. *In vitro* effects of allicin on the production of QS‐controlled virulence factors and biofilm formation in *P. aeruginosa* were assessed using standard assays and fluorescence microscopy, respectively, by Lihua *et al*. (2013). The findings indicated that allicin (128 µg ml^‐1^) inhibited early bacterial adhesion up to 9 h following treatment, while EPS production was reduced by over threefold in a dose‐dependent manner. Biofilm architecture also underwent a transition to a thinner and looser form. The production of exotoxin A and elastase was downregulated by threefold and 10‐fold, respectively, while pyocyanin and rhamnolipid production was completely abrogated under subinhibitory concentration of allicin. Lately, the mechanisms underlying allicin‐mediated virulence attenuation in *P. aeruginosa* were unearthed (Xu *et al*., 2019). It was revealed that allicin inhibited the production of pyocyanin (*phzB2* and *phzM* genes), pyoverdine, elastase and rhamnolipids (*rhlA*, *rhlB* and *rhlC* genes) in a concentration‐dependent manner. Concisely, qRT‐PCR analysis showed prominent inhibition of *rhlI*, *pqsA*, *pqsD and pqsR* transcripts, without affecting the *las* system. The suppression of *rhl* and *pqs* systems was substantiated when supplementation with C4‐HSL and PQS rescued the pathogenic phenotype of *P. aeruginosa* PAO1 strain by restoring the production of virulence factors. These studies have established the ethnopharmacological importance of allicin as a therapeutic drug that can be exploited to disarm the QS‐dependent virulence determinants of *P. aeruginosa*.

### Terpinen‐4‐ol

Terpinen‐4‐ol is a naturally occurring monoterpene, an isomer of α‐terpineol that forms the principle bioactive in tea tree oil. Thyme essential oil containing numerous phytochemicals, including terpinen‐4‐ol, has been shown to suppress QS, thereby reducing twitching motility and pyocyanin production (~ 74%) in *P. aeruginosa* (Bukvički *et al*., 2016). The terpene alcohol negated biofilm formation and exhibited prolific anti‐quorum activity by inhibiting the production of violacein (> 90%) in the AHL‐mediated QS reporter strain, *C. violaceum* (Kerekes *et al*., 2013). Further, Noumi *et al*. (2018) examined the QQ potential of terpinen‐4‐ol on swarming motility in PAO1. Migration capabilities of the PAO1 strain were majorly inhibited by 25% in the presence of terpinen‐4‐ol. Moreover, this monoterpene was also shown to abrogate biofilm production and simultaneously inhibit the adhesion of *Staphylococcus aureus* to polystyrene and glass surfaces.

Most recently, an elaborate investigation shed light on the potential of terpinen‐4‐ol to attenuate QS pathways, virulence determinants and biofilm formation in *P. aeruginosa* (Bose *et al*., 2020a). The QQ ability of terpinen‐4‐ol was assessed *in silico* with QS receptors, providing strong evidence for its interaction with LasR, RhlR and PqsR. Subinhibitory levels of terpinen‐4‐ol were found to downregulate the expression *of lasI*, *lasR*, *rhlI*, *rhlR*, *rhlAB*, *lasB*, *aprA*, *toxA* and *plcH* QS transcripts in *P. aeruginosa* PAO1 strain. Further, the production of alginate, elastase, haemolysin and pyocyanin was found to be significantly reduced along with swimming, swarming and twitching motilities upon treatment with terpinen‐4‐ol, corroborating the findings of virulence gene expression profile. Interestingly, biofilm architecture was disrupted leading to fragile biofilm with significantly reduced bacterial adherence. Nevertheless, a novel combinational approach using terpinen‐4‐ol and ciprofloxacin proved to act synergistically against *P. aeruginosa* and its associated virulence factors, paving way for the innovation of novel anti‐virulence therapies.

### Zingerone

Zingerone, also called vanillylacetone, is another bioactive derived from ginger responsible for imparting a sweet flavour. Researches over the years have proclaimed its diverse pharmacological properties as a potent anti‐inflammatory, anti‐diarrhoeic and anti‐diabetic agent (Ahmad *et al*., 2015b). Nonetheless, the use of zingerone for silencing QS mechanisms and negating biofilm formation in *P. aeruginosa* has been assessed by various investigators. Supplementation with zingerone at sub‐MICs impeded swimming, swarming and twitching motilities by more than 50% as compared to its absence (Kumar *et al*., 2013). SEM analysis revealed that zingerone prevented biofilm formation by *P. aeruginosa* on catheter surface without affecting bacterial planktonic growth. Also, a combination of ciprofloxacin and zingerone was shown to completely eradicate preformed biofilm of *P. aeruginosa* at significantly higher rates. In a follow‐up study, Kumar *et al*. (2015) demonstrated that zingerone drastically truncated the production of QSSMs: C12‐HSL, C4‐HSL and PQS in clinical isolates of *P. aeruginosa* and standard strain PAO1. In consequence, the production of virulence factors such as pyocyanin, haemolysins, elastases, proteases and rhamnolipids was also inhibited. This QQ capability of zingerone was adjudged by *in silico* studies, where its affinity towards QS receptors was determined. Interestingly, molecular docking showed profound interaction between zingerone and receptor binding pockets of LasR, RhlR and PqsR attributed to the inhibition of these QS systems in *P. aeruginosa*.

In an attempt to enhance drug delivery with high specificity and efficacy, the development of nanoformulations is on the move. In this regard, zingerone‐loaded chitosan nanoparticles (Z‐NPs) have been developed with promising anti‐virulence properties against *P. aeruginosa*. The Z‐NPs were reported to be stable under varying temperatures, inhibiting the production of QSSMs in *P. aeruginosa* using a reported assay employing *Agrobacterium tumefaciens* A136 strain (Sharma *et al*., 2020b). The production of alginate, elastases, haemolysins, proteases and pyocyanin was drastically reduced when *P. aeruginosa* was passaged under sub‐MIC of Z‐NPs as compared to zingerone alone. Moreover, all the bacterial motilities were inhibited by more than fourfold, while qRT‐PCR studies indicated that expression of *lasR*, *rhlI* and *rhlR* was downregulated by more than twofold in the presence of Z‐NP at sub‐MIC level. As a result, an anti‐biofilm response was observed with Z‐NPs, which successfully prevented biofilm formation and eradicated preformed biofilms of *P. aeruginosa*. Recently, the *ex vivo* and *in vivo* efficacies of Z‐NPs were examined against *P. aeruginosa* in a murine model of acute pyelonephritis (Sharma *et al*., 2020a). Z‐NPs were reported to be nontoxic to HEK‐293 cells (~ 94% survival rate), reducing the pathogenicity of *P. aeruginosa* and ensuring the elimination of bacteria from renal tissues with better recovery. Additionally, Z‐NPs enhanced the serum bactericidal property, accelerated the phagocytic uptake and killing of *P. aeruginosa* cells by mouse macrophages *ex vivo* and induced an anti‐biofilm response *in vitro*. These evidences have outlined the significance of zingerone as an alternative to antibiotics in combating pseudomonal infections.

### Curcumin

Curcumin is the major bioactive present in *Curcuma longa* (turmeric) imparting a bright yellow colour to the modified stem. It is extensively used for food flavouring and colouring, and as a cosmetic ingredient. Apart from possessing broad‐range antimicrobial activity, curcumin has been reported to exhibit profuse QQ property against *P. aeruginosa*. Studies pioneered by Rudrappa and Bais reported that sub‐MICs of curcumin (3 µg ml^‐1^) inhibit biofilm formation, production of QSSMs and QS‐regulated virulence elements including protease (50%), elastase (50%) and pyocyanin (~ 70%) in PAO1 (Rudrappa and Bais 2008). Supplementing exogenous curcumin significantly prolonged survival rates and rescued killing in PAO1‐infected *C. elegans*. These outcomes were substantiated by transcriptome analysis, which indicated the downregulation of 31QS genes in the presence of curcumin sub‐MICs. Similarly, curcumin displayed anti‐QS activity by inhibiting violacein production by ~ 89% in the *C. violaceum* biosensor (Packiavathy *et al*., 2014). At 100 µg ml^‐1^, curcumin effectively dislodged pseudomonal biofilms by 89%, retarding swimming and swarming motilities of *P. aeruginosa* by sevenfold and fivefold, respectively, alongside decreasing the production of alginate and rhamnolipids by more than 50%.

In an attempt to target *P. aeruginosa*, the synergistic effect of curcumin‐natural honey (ChC) mixture on QS and virulence factors was examined *in vitro* (Jadaun *et al*., 2015). ChC significantly lowered the production of C12‐ and C4‐AHLs, alginate, rhamnolipids, haemolysins, pyocyanin, pyoverdine, pyochelin, LasB elastase and LasA protease than honey or curcumin alone. Using an enzyme‐coupled reporter assay, the expression of pivotal *las* and *rhl* QS genes was reduced by more than 60% and 50% respectively. Interestingly, biofilm formation was completely abolished with ChC, thereby increasing the susceptibility of bacterial cells to carbapenems and cephalosporins. In the same direction, Bahari *et al*. (2017) showed that supplementing curcumin at sub‐MIC levels during bacterial growth drastically reduced the MICs of azithromycin and gentamicin against *P. aeruginosa*. The synergistic interaction between curcumin at sub‐MICs and antibiotics also quenched QS signals, resulting in downregulation of *las* and *rhl* genes, accompanied by a reduction in the swarming and twitching motilities and biofilm formation capability of PAO1. Also, nanocomposites of zinc–curcumin have been developed to enhance the delivery and bioavailability of this QQ bioactive inside *P. aeruginosa* (Prateeksha *et al*., 2019). The nanoformulations were reported to vanquish LasR and RhlR QS systems, thereby protecting PAO1‐infected mice and lung epithelial cells by diminishing the expression of virulence factors, biofilm formation without hindering bacterial growth. Moreover, curcumin‐coated medical implants have been developed to combat nosocomial infections. Coating of curcumin‐loaded gold nanoparticles on surgical sutures and chitosan–curcumin complexes on stainless‐steel substrates showed promising antimicrobial activities with high stability and sustained release of curcumin (Sunitha *et al*., 2017; Virk *et al*., 2019). Therefore, curcumin can be exploited as a potent bioactive against *P. aeruginosa*, laying a strong foundation for its application in anti‐virulence therapy.

### Cinnamaldehyde

Cinnamaldehyde, derived from *Cinnamomum*, is a phenylpropanoid and the major component of cinnamon oil that imparts the spice its distinct flavour and aroma. In preliminary studies, it was shown to effectively inhibit the AHL‐mediated QS systems of *Vibrio harveyi* and biofilm formation in *P. aeruginosa* (Niu and Gilbert 2004; Niu *et al*., 2006). An elaborate study has unravelled the QQ potential of cinnamon oil *in vitro* (Leoni *et al*., 2015). The authors indicated that cinnamon oil quenched the synthesis of AHLs, thereby inhibiting biofilm formation, swarming motility and production of pyocyanin, alginate and proteases in PAO1. In the same light, sub‐MICs of cinnamaldehyde were reported to inhibit swarming motility and disrupt pseudomonal biofilms by lowering the levels of intracellular cyclic di‐GMP (Topa *et al*., 2018). A breakthrough study by Ahmed and colleagues reported the anti‐QS response of trans‐cinnamaldehyde at sub‐MIC levels against *P. aeruginosa* (Ahmed *et al*., 2019). Cinnamaldehyde (2.2 mM) stimulated the downregulation of *las* and *rhl* QS systems, particularly *lasR* and *lasI* transcripts by sevenfold and 13‐fold, respectively, during the stationary phase of bacterial growth. This accounted for the reduced production of elastase, pseudomonal biofilms, pyocyanin and proteases by 22%, 26%, 32% and 65%, respectively, and repressed rhamnolipid expression (*rhlA*) by 100‐fold in PAO1. Further, the combinational effect of cinnamaldehyde and antibiotics on QS was evaluated recently (Topa *et al*., 2020). Synergism was confirmed with colistin (as antimicrobial) and tobramycin (as QSI). Cinnamaldehyde (1.5 mM) alone induced the repression of *lasB*, *rhlA* and *pqsA* genes by ~ 50, 60% and 45% respectively. The effect was further enhanced in the presence of tobramycin by ~ 70% for the three test genes. On the contrary, combinational effect with colistin and tobramycin enhanced the anti‐biofilm efficacy by 75% and 84%, respectively, compared with cinnamaldehyde alone (~ 30%). Additive activities also accelerated the dispersion of preformed PAO1 biofilms by ~ 90% in case of both antibiotics. Interestingly, cinnamaldehyde‐encapsulated chitosan nanoparticles at sub‐MIC levels were developed to attenuate QS and biofilm formation in *P. aeruginosa* (Subhaswaraj *et al*., 2018). The nanoformulations were shown to hinder the swimming and swarming motilities of PAO1 with notable inhibition of pyocyanin, LasA protease, EPS, biofilm and rhamnolipid production as compared to cinnamaldehyde alone. Since this bioactive exhibits magnificent QQ activity, it is most likely to be the game‐changer towards the fight against this notorious pathogen.

### Eugenol

Eugenol is a phenolic component obtained from various plant products, majorly spices, herbs and oils. Its broad‐range antimicrobial properties have been known for years, while its QQ ability has recently surfaced in the last decade. Eugenol was identified as the primary component in clove oil responsible for inhibiting QS‐controlled gene expression (Zhou *et al*., 2012). Sub‐MICs of eugenol (200 µM) were reported to diminish violacein production (~ 50%) in *C. violaceum* f026 biosensor along with QS‐controlled virulence factors: elastases (~ 32%), pyocyanin (~ 60%) and biofilm formation (~ 36%). These downstream effects were reported to be an outcome of *las* and *pqs* QS system inhibition by eugenol. Similarly, sub‐MICs of methyl eugenol (30 μg ml^‐1^) were shown to inhibit the swimming (~ 66%) and swarming (~ 24%) motilities, and biofilm formation in PAO1 by 90% (Packiavathy *et al*., 2012). Another study affirmed the eugenol‐mediated attenuation of PAO1 QS system and QS‐associated virulence elements by abrogating the synthesis of QSSMs (C12‐HSL, C4‐HSL) and directly repressing the transcription of *lasR*, *lasI*, *rhlR* and *rhlI* QS genes (Rathinam *et al*., 2017). *In silico* analysis coupled with *in vitro* reporter assays confirmed the competitive binding of eugenol to the LasR QS receptor, thereby driving the repression of QS‐regulated genes. Recently, eugenol nanoemulsions were developed by Lou *et al*., (2019) with high QQ efficacy against *P. aeruginosa*, repressing the transcription of *lasI* and *rhlI* genes and inhibiting the production of violacein, pyocyanin, rhamnolipids and pseudomonal biofilms at a significantly higher rate than eugenol alone.

### Citral

Citral is a terpenoid that constitutes about 70–80% of lemongrass oil and imparts the herb its characteristic aroma and taste. Preliminary investigations using reporter assays have pointed towards the inhibition of C12‐HSL‐mediated QS systems of *P. putida* by citral at relatively higher concentrations (Colorado *et al*., 2012). Citral has been reported to inhibit violacein production by 96.18% in *C. violaceum* biosensor, thereby asserting its role as potent QSI (Rueda and Salvador 2020). For *P. aeruginosa*, sub‐MIC of citral (840 µg ml^‐1^) was shown to inhibit swarming motility by 93% with a significant reduction in the levels of pyoverdine production. For enhanced efficacy against QS and biofilm formation, various derivatives of citral (CD1, CD2 and CD3) have been developed and tested *in vitro*. These derivatives abrogated biofilm production and downregulated the expression of QS genes in *C. violaceum*, without altering bacterial growth (Batohi *et al*., 2021). This can serve as a template that can be tested against QS systems and virulence factors of *P. aeruginosa*.

Interestingly, a study focused on enhancing citral's solubility and QQ potential by synthesizing its glycomonoterpene *via* conjugation with glucose (Patil *et al*., 2016). The conjugate was shown to abolish QS by inhibiting the production of violacein, pyoverdine and pseudomonal biofilms at significantly lower concentrations than citral itself, signifying enhanced bioavailability and delivery of citral to attenuate the virulence of *P. aeruginosa*. A virtual screening study recently identified citral as a potential QSI against *P. fluorescens* (Ding *et al*., 2019). Molecular docking revealed that citral exhibited a high docking score (121.52) with the active site LuxR receptor and speculated this interaction to be stabilized *via* hydrogen bonds, carbon‐hydrogen bonds and π‐alkyl bonds. *In vitro* experiments showed that citral exhibited promising anti‐QS activities against *P. fluorescens*, thereby inhibiting the swimming motility, biofilm formation, and production of extracellular proteases and siderophores, without hindering bacterial growth. However, the precise mechanism that citral employs for QQ remains to be explored.

### Coumarins

Coumarins are secondary plant metabolites that constitute a large family of fused benzene and pyrone rings. Few coumarins that exhibit antimicrobial activity have also been regarded as phytoalexins, produced in response to pathogenic infection. Furocoumarins present in grapefruit juice were shown to repress the activities of AI‐1 and AI‐2 by more than 95% in *V. harveyi* reporter strain indicating their anti‐QS potential (Girennavar *et al*., 2008). Moreover, biofilms of *P. aeruginosa* were inhibited by 18% and 27% by furocoumarins dihydroxybergamottin and bergamottin, respectively, without altering bacterial growth. In a virtual screening study, the coumarins, aesculetin and aesculin, were reported to interact with the signal‐binding domain of TraR protein and as inhibitors of biofilm formation in *P. aeruginosa* (Zeng *et al*., 2008). For the first time, the QQ abilities of coumarin and its hydroxylated derivatives were illustrated against *P. aeruginosa in vitro* (D'Almeida *et al*., 2017). 3‐Hydroxycoumarin, 7‐hydroxycoumarin and 6,7‐dihydroxycoumarin were shown to inhibit violacein production in *C. violaceum*, while the latter two reduced pseudomonal biofilm formation at significantly higher rates than coumarin. The elastolytic activity of *P. aeruginosa* was also reduced upon exposure to coumarin and its derivatives. Recently, the precise mechanism of coumarin‐mediated QQ in *P. aeruginosa* was unearthed (Zhang *et al*., 2018). Coumarin downregulated the expression of key QS genes: *lasI*, *rhlI*, *rhlR*, *pqsB*, *pqsC*, *pqsH* and *ambBCDE*, leading to attenuation of QS and inhibition of protease and pyocyanin production. Interestingly, coumarin led to the disruption of cyclic di‐GMP signalling by lowering its cellular levels by 36.4% compared with 69.7% in its absence, thereby inhibiting biofilm formation in the wound model *in vitro*. These events stimulated by coumarin cumulatively led to the attenuation of virulence in *P. aeruginosa*.

### Carvacrol

The monoterpenoid phenol, carvacrol or cymophenol is derived from the oregano plant and bears a warm, pungent odour. Early investigations on carvacrol showed that its sub‐MICs inhibit QS and biofilm production in *C. violaceum ATCC 12472*, a reporter strain used for detecting anti‐QS compounds (Fakunle *et al*., 2013; Burt *et al*., 2014). However, these findings were extended to *P. aeruginosa* and substantiated with subsequent experimentations (Rodriguez *et al*., 2017). The authors indicated that carvacrol at concentrations ranging between 0.9 and 7.9 mM inhibited *P. aeruginosa* biofilm formation on stainless steel by 1.5–3 log CFU cm^‐2^. Moreover, carvacrol at 0.7 and 3.9 mM was shown to significantly inhibit violacein production (~ 50%) and pyocyanin (~ 60%), respectively, without affecting bacterial growth and viability. These findings can be accounted for the use of carvacrol at comparatively higher concentrations. Lately, the anti‐QS prospects of carvacrol against *P. aeruginosa* were investigated by standard assays *in vitro* and through docking studies (Rodriguez *et al*., 2019). The production of C4‐HSL, C6‐HSL and C12‐HSL was lowered by carvacrol in a concentration‐dependent manner, with 1.9 mM reducing their synthesis by 63%, 80%, and 60% respectively. Carvacrol was reported to completely repress the transcription of *lasR* gene, without affecting *lasI* gene. Consequently, the QS‐mediated virulence factors such as swarming motility, biofilm formation and production of pyocyanin, LasA protease and LasB elastase were diminished (Rodriguez *et al*., 2019). The findings were further substantiated when carvacrol was reported to interact with the amino acid residues of LasR and LasI active domains, repressing *lasR* and inhibiting LasI activity. This establishes its potency as a novel quorum quencher against pseudomonal virulence factors at both enzymatic and gene levels.

### Baicalein

Baicalein is a trihydroxyflavone derived from the roots of *Scutellaria baicalensis*, renowned for its use as a traditional herbal medicine with antioxidant and antimicrobial activity. It has been shown to attenuate virulence in *S. aureus* and *E. coli* by abrogating QS mechanisms and biofilm formation *in vitro* (Chen *et al*., 2016; Peng *et al*., 2019). Recently, baicalein at subinhibitory concentrations has been elucidated to attenuate the QS circuitry in *P. aeruginosa*. The synthesis of QSSMs, C4‐HSL and C12‐HSL was lowered by twofold along with significant downregulation of both *las* and *rhl* QS genes (Seleem *et al*., 2017). This resulted in the global attenuation of virulence factors such as swimming, swarming and twitching motilities, biofilm formation, and the production of alginate, LasB elastase, LasA protease, pyocyanin and rhamnolipids. Interestingly, the production of inflammatory cytokines, IL‐1β, IL‐6, IL‐8 and TNF‐α, in *P. aeruginosa*‐infected macrophages was also reduced significantly alongside suppression of MAP kinase and NF‐κB signalling pathways (Luo *et al*., 2016). This indicated baicalein’s potential as an anti‐virulence drug against *P. aeruginosa*. In a similar study, Luo *et al*. (2016) illustrated the effects of baicalein sub‐MICs on QS and virulence factors *in vitro* alongside *in vivo* investigations with *P. aeruginosa*‐infected *C. elegans* and murine model of peritoneal implant. Baicalein was reported to repress the expression of pivotal QS‐regulatory genes, including *lasI*, *lasR*, *rhlI*, *rhlR*, *pqsA* and *pqsR*, thereby limiting the production of QSSMs resulting in the downregulation of global virulence machinery and biofilm formation of *P. aeruginosa in vitro*. Baicalein‐treated *C. elegans* showed greater survival rates, while it accelerated the clearance of bacterial cells from the implants of infected mice compared with the untreated group. Administration of baicalein instigated inflammatory responses *via* activation of Th1‐induced immune response to enhance the elimination of pathogen, augmenting its anti‐QS agent and anti‐biofilm potential.

### Naringenin and naringin

Naringenin is a colourless flavone, an integral bioactive component of citrus fruits, predominantly grapefruit. Its O‐glycosylated derivative, naringin, is another principle phytochemical that imparts bitterness to citrus fruits. Both these flavones have been reported to exhibit QQ activities against *P. aeruginosa*. Naringenin was first reported to quell the production of QS‐regulated virulence factors of *P. aeruginosa* (Vandeputte *et al*., 2011). The production of elastase and pyocyanin was inhibited by 46% and 87% in the presence of naringenin respectively. This reduction occurred by the virtue of downregulated *lasI*, *lasR*, *rhlI*, *rhlR* and *lasB* expression, sharply declining the production of QSSMs, C4‐HSL and C12‐HSL in naringenin‐treated cultures of *P. aeruginosa in vitro*. Intriguingly, QS inhibition by naringenin was not limited to the reduction in QSSMs but also related to the defective functioning of the *RhlR*‐C4‐HSL complex. A study pointing towards the precise molecular mechanism of naringenin‐mediated QQ has recently surfaced. It was revealed that naringenin outcompeted C12‐HSL for binding to its cognate LasR receptor in a time‐dependent manner (Amado *et al*., 2020). LasR inhibition was heightened when naringenin was directly associated with the nascent receptor form than its activated form. Amusingly, naringenin disrupted the expression of QS genes and QS‐regulated virulence genes during the early phase of bacterial growth, while the inhibitory effect was lost upon transition to the stationary phase. This suggested that competition between C12‐HSL and naringenin for the LasR receptor is a function of bacterial growth.

Naringin has also been shown to exert similar anti‐QS effects. It was initially demonstrated to disarm the QS machinery in the enteric pathogen, *Yersinia enterocolitica*, by inhibiting swimming and swarming motilities along with biofilm formation (Truchado *et al*., 2012). Naringin has recently been shown to effectively eradicate pseudomonal biofilms on inanimate and catheter surfaces, inhibiting swimming motility and production of EPS matrix (Dey *et al*., 2020). In contrast, naringin's anti‐biofilm and anti‐virulence efficacy against *P. aeruginosa* was appreciably augmented upon supplementation with antibiotics, ciprofloxacin and tetracycline compared with their solo treatment. These evidences establish that both naringenin and naringin are potential QQ and anti‐biofilm agents against *P. aeruginosa*.

### Quercetin

Quercetin is a pentahydroxyflavone recovered from pericarps of fruits, seeds and leafy green vegetables with extensive anti‐inflammatory, antioxidant and anti‐cancerous properties. Early investigations reported the ability of quercetin at sub‐MICs to impede biofilm formation (~ 95%) and twitching motility in *P. aeruginosa* (Pejin *et al*., 2015). The adverse effect of quercetin at sub‐MICs and its synergism with various antibiotics (at sub‐MIC) against pseudomonal biofilms has also been elucidated (Vipin *et al*., 2019). Several studies on this aspect have discretely pointed towards the QQ potential of quercetin, abrogating the virulence determinants of *P. aeruginosa*. A breakthrough study indicated that quercetin did not hinder bacterial growth but triggered the inhibition of biofilm formation (50%) and the production of elastase, protease and pyocyanin significantly (Ouyang *et al*., 2016). Quercetin at 16 µg ml^‐1^ led to the notable repression of essential QS genes including *lasI*, *lasR*, *rhlI* and *rhlR* by 34%, 68%, 57% and 50% respectively. This laid down the foundation for exploring quercetin as a potential therapeutic.

Further concrete evidences have been produced to prove this point from multiple investigations by independent research groups. Quercetin at sublethal concentrations (5–40 μg ml^‐1^) inhibited the production of alginate by 9–65%, bacterial biofilms by 8‐80% and EPS matrix by 15–73% (Gopu *et al*., 2015). Swimming and swarming motilities were reduced significantly with quercetin (80 µg ml^‐1^) by 50% and 75% respectively. Molecular docking of LasR receptor with quercetin revealed strong interactions, abrogating QS by a potential change in the receptor conformation upon quercetin binding. Quecan *et al*. (2019) reported the QQ potential of quercetin aglycone and quercetin 3‐β‐D‐glucoside against *P. aeruginosa*. The bioactives inhibited swarming motility and production of violacein *in vitro*, while biofilm formation remained unaffected. However, *in silico* analysis suggested effective binding of the two quercetins with the AHL‐binding domain of LasR receptor of PAO1, providing a rationale for its anti‐virulence effects. It has also been proposed that quercetin retards biofilm formation in *P. aeruginosa via* inhibition of *vfr* and biofilm‐related genes (*pelA*, *pslA*), regardless of the presence of QS genes (Ouyang *et al*., 2020). This was speculated to be a consequence of *lasI*/*lasR* inhibition by quercetin, affecting the expression of *vfr* gene, resulting in abrogation of pseudomonal biofilm. Thus, quercetin represents a promising candidate that requires precise evaluation in animal models for its deployment in anti‐virulence therapy against *P. aeruginosa*.

### Caffeine

Caffeine, or 1,3,7‐trimethylxanthine, is a natural ingredient in beverages such as coffee and tea and is often proclaimed for its CNS stimulation and antidepressant properties. Although caffeine has been in use for centuries since its discovery in 1819, its anti‐QS potential was revealed in the last decade. The inceptive study reported caffeine to impede violacein production in *C. violaceum* in a dose‐dependent manner without exerting any antibacterial activity (Norizan *et al*., 2013). Caffeine did not stimulate the degradation of AHLs but reduced the violacein production induced by PAO1 AHLs indicating its role in repealing the synthesis of short‐chain AHLs (C6‐HSL) in PAO1. Swarming motility of PAO1 was also inhibited by caffeine (300 µg ml^‐1^) with short and undefined tendrils. In a similar study, caffeine was identified as the major constituent of fenugreek seed extract responsible for QQ activities (Husain *et al*., 2015). At 200 µg ml^‐1^, caffeine reduced violacein production by 87% in *C. violaceum* and diminished elastase, protease and pyocyanin production by 68%, 78% and 74% in PAO1 respectively. Caffeine was also shown to inhibit swarming motility (70%) and biofilm formation (64%) in PAO1. The latest study on caffeine’s potential to target QS corroborated the previous findings using bench‐based experiments and *in silico* studies. It was confirmed that caffeine at sub‐MIC levels (80 μg ml^‐1^) inhibited the production of proteases (~ 65%) and pyocyanin (~ 60%), retarded biofilm formation by 50%, and disrupted swarming motility at significant rates in *P. aeruginosa* (Chakraborty *et al*., 2020). Additionally, molecular docking analysis confirmed these findings by suggesting strong interactions between caffeine and LasR, LasI proteins attributing this bioactive as a potential anti‐QS compound.

**Fig. 3:**
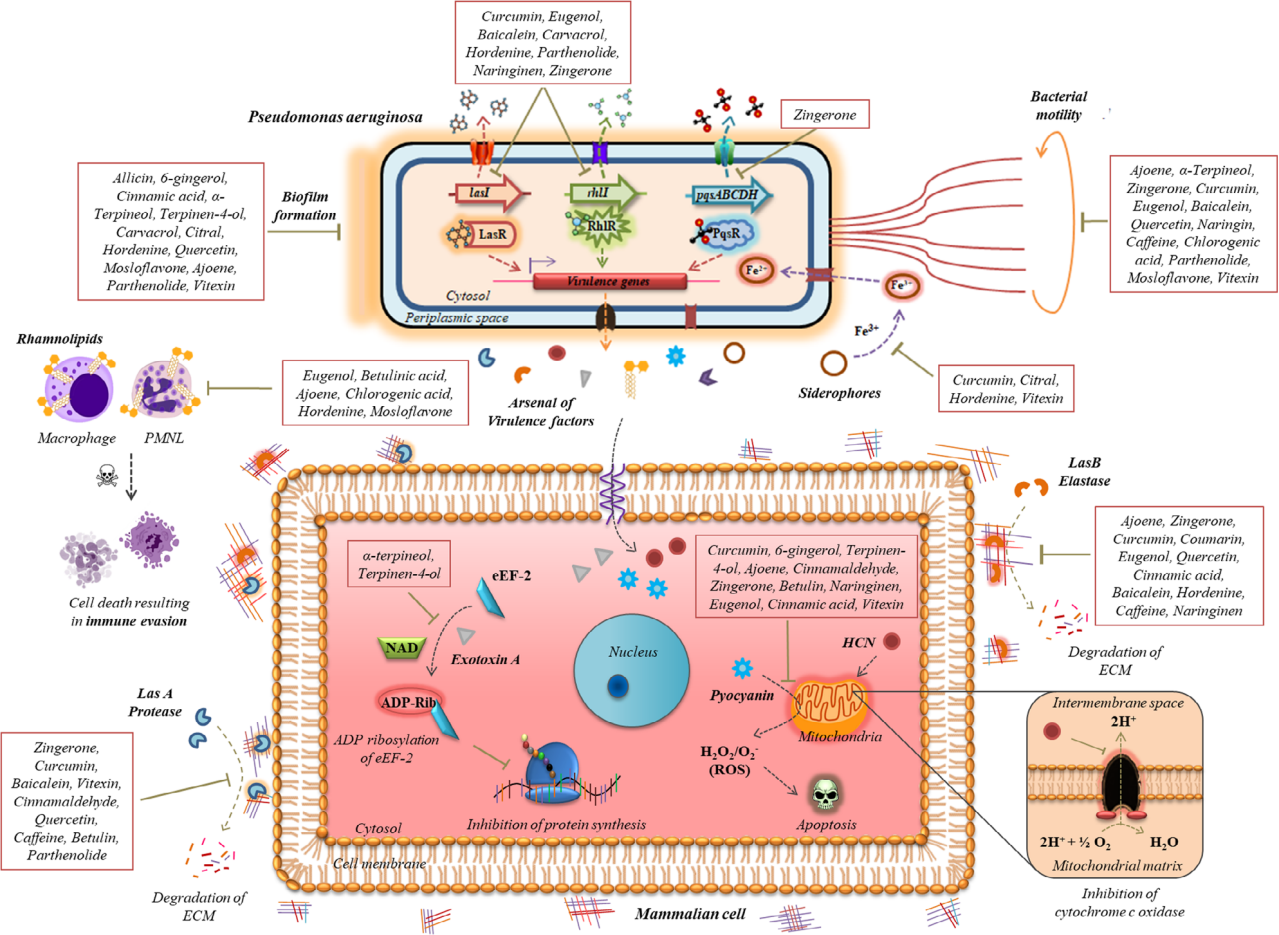
Mechanistic depiction of *P. aeruginosa* QS‐regulated virulence factors that alter host physiology and how phytochemicals attenuate QS and target virulence at different stages. Plant bioactives (in boxes) have been reported to attenuate different QS systems, inhibiting QS‐regulated virulence factors in *P. aeruginosa*. The figure was created using CorelDRAW Graphics Suite 12 based on the experimental evidences cited in this review.

Apart from the phytochemicals mentioned above, other bioactives have also been shown to possess QQ and anti‐virulence properties. Findings related to these bioactive phytochemicals are summarized in Table 1. However, these account for preliminary evidences and require further validation to develop them into potential anti‐QS agents. In summary, investigations have reported noteworthy findings for repurposing plant‐derived bioactives as an anti‐virulence therapy against *P. aeruginosa*. To epitomize the anti‐virulence nature of phytochemicals as per the scientific explorations cited in this review, a mechanistic overview illustrating the virulence machinery of *P. aeruginosa* and how phytochemicals target them is depicted in Fig. 3.

**Table 1:** Studies showing preliminary evidence that bioactive phytochemicals demonstrate curative potential against quorum sensing and virulence factors in *P. aeruginosa*.

Name of bioactive phytochemical	Effects observed against QS and various virulence factors of *P. aeruginosa*									References
	Alginate production	LasB Elastase	LasA protease	Pyocyanin production	Pyoverdine production	Bacterial motility	Biofilm formation	Rhamnolipid production	Other remarks	
Betulin (125 µg ml^‐1^)	↓~ 88%	↓~ 17%	↓~ 74%	↓~ 75%		Swarming motility ↓ ~ 51%	↓~ 57% with EPS matrix ↓ ~ 31% and ↓ ~ 20%	↓~ 20%	Mortality in infected *C. elegans* ↓ ~ 12%. Molecular docking suggested interactions with LasR and RhlR QS receptors	(Rajkumari *et al*., 2018a)
Betulinic acid (125 µg ml^‐1^)	↓~ 55%	↓~ 8%	↓~ 84%	↓~ 75%		Swarming motility ↓ ~ 47%	↓~ 33% with EPS matrix ↓ ~ 36% and eDNA release ↓ ~ 20%	↓~ 22%	↑Survival rate in infected *C. elegans* by ~ 21%. Moderate interactions with RhlR by *in silico* analysis	(Rajkumari *et al*., 2018a)
Chlorogenic acid (2.56 mg ml^‐1^)		↓~ 38%	↓~ 40%	↓~ 25%		Swarming motility ↓ ~ 33%	↓~ 52%	↓~ 30%	↓expression of QS genes: *lasI* 85.09%, *lasR* 48.63%, *rhlI* 27.98%, *rhlR* 34.7%, *pqsA* 73.08%, and *pqsR* 45.85%. Survival rate in infected *C. elegans* ↑ ~ 16%. ↑ Wound closure in infected mouse model by ~ 84% with reduced bacterial load	(Wang *et al*., 2019)
Cinnamic acid (250 µg ml^‐1^)	↓~ 50%	↓~ 71%	↓~ 22%	↓~ 81%		Swarming motility ↓ ~ 42%	↓~ 50% with eDNA release ↓ ~ 25% and EPS production ↓ ~ 32%. Biofilm thickness ↓ 12 µm	↓~ 17%	Survival rate ↑ in infected *C. elegans* by ~ 59%. Strong binding towards LasR and RhlR QS receptors by *in silico* studies	(Rajkumari *et al*., 2018b)
Hordenine (750 µg ml^‐1^)	↓~ 50%	↓46%	↓~ 40%	↓~ 48%	↓~ 42%	Swimming motility ↓ ~ 50% and swarming motility ↓ > 50%	↓26%. With netilmicin, biofilm ↓ ~ 52% and preformed biofilms ↓ 43%	↓~ 40%	↓QS Signals; C4‐HSL ↓ 74%, C12‐HSL ↓ 40%. Transcript levels of *las* and *rhl* ↓ ~ 50%	(Zhou *et al*., 2018)
Mosloflavone (125 µg ml^‐1^)	↓~ 57%	↓~ 36%		↓~ 60%		Swimming and swarming motility ↓ > 50%	↓~ 52% and EPS production ↓ ~ 47%	↓~ 34%	↓QS Gene and QS‐target transcripts: *lasI* 61%, *lasR* 92%, *rhlI* 57%, *rhlA* 48%, *rhlR* 22%, *lasB* 38%, *toxA* 61%, *aprA* 58% and *exoS* 78%. Survival rate ↑ in infected *C. elegans* by 37%	(Hnamte *et al*., 2019)
Parthenolide (1 mM)			↓~ 45%	↓~ 35%		Swarming motility ↓ > 80%	↓~ 56% with marked reduction in EPS matrix		C12‐HSL levels ↓ 53%. QS Genes expression ↓ *lasI* 45%, *lasR* 57%, *rhlI* 39%, *rhlR* 51%. High affinity towards RhlR receptor from *in silico* studies	(Kalia *et al*., 2019)
Taxifolin (4 mM)		↓~ 47%		↓~ 56%					↓of QS genes: *lasI*, *lasR*, *rhlI* and *rhlR* by threefold, 2.5‐fold, 1.3‐fold and 4.3‐fold respectively	(Vandeputte *et al*., 2011)
Vitexin (110 µg ml^‐1^)		↓~ 38%	↓~ 39%	↓~ 25%	↓~ 25%	Swarming motility ↓ > 60%	↓~ 56% with ↓ ~ EPS matrix		↓~ 1.4 log reduction in bacterial load in liver and spleen tissues, and on implanted catheter recovered from murine model	(Das *et al*., 2016)

Down arrow (↓) indicates the significant reduction in the levels of particular virulence factor tested. Upward arrow (↑) indicates the significant increase in the particular attribute.

## Other inhibitors of *P. aeruginosa* QS systems

Apart from bioactive phytochemicals, different QSIs have been identified, ranging from antibiotics to synthetic drugs (analogs) and even microbial enzymes. These have been widely explored for their QQ potential in medical and biotechnological arenas (Kalia *et al*., 2019). Antibiotics such as azithromycin (Tateda *et al*., 2001) and ciprofloxacin (Gupta *et al*., 2016) have been reported for their QQ and anti‐virulence properties against *P. aeruginosa*. Even niclosamide (anthelminthic) and itaconimides (antifungal) have been repurposed for their QQ abilities to disarm the virulence factors in *P. aeruginosa* (Imperi *et al*., 2013; Fong *et al*., 2019). Also, the anti‐QS activities of compounds such as salicylic acid and sodium ascorbate have been documented with a monumental decrease in biofilm formation and the expression of QS genes in PAO1 (El‐Mowafy *et al*., 2014; Ahmed *et al*., 2019). With advancements in structural biology and structure–activity relationships, a plethora of synthetic compounds, including novel conjugates and drug analogs with QQ quenching abilities, have been developed over the last two decades. *In silico* analysis accompanied by bench‐based investigations with novel thiazolidinedione‐type compounds has been shown to significantly reduce QSSM production and biofilm formation in *P. aeruginosa* without hindering its growth (Lidor *et al*., 2015). Methyl anthranilate, an analog of anthranilate moiety of 2‐heptyl‐3‐hydroxy‐4‐quinolone, has been reported for repressing the production of PQS and LasB elastase in PAO1 under sublethal concentrations (Calfee *et al*., 2001). Similarly, halogenated anthranilate analogs inhibited PQS biosynthesis and PQS‐driven gene expression, and restricted the systemic dissemination of *P. aeruginosa* in a burn wound model (Lesic *et al*., 2007). *N*‐Decanoyl cyclopentylamide has also been reported to attenuate QS and virulence in PAO1 by abrogating the interaction between LasR and RhlR response regulators and their autoinducers (Ishida *et al*., 2007). Conjugate compounds consisting of an antibiotic or phytochemical chemically linked to a QSI have also been synthesized and shown to augment the QQ activities of the QSI against *P. aeruginosa*. Recently, a chemically synthesized LasR QSI conjugated with ciprofloxacin (ET37) has been demonstrated to decrease the production of QS‐associated pyocyanin and elastases, biofilm formation and antibiotic tolerance in clinical isolates of *P. aeruginosa* (Bortolotti *et al*., 2019). This approach has been further extended in the form of an emerging ‘Trojan Horse’ strategy that employs the conjugation of a potent drug with either antibiotic(s) or pathogen‐encoded virulence factors for deceitful drug delivery. In this context, a novel pyochelin–zingerone conjugate has been recently developed to target the QS circuitry and virulence in *P. aeruginosa* (Nosran *et al*., 2021). The conjugate reportedly mimicked the function of pyochelin and successfully associated with its cognate receptor (FptA), allowing its entry into the pathogen by deception and attenuating QS circuits, biofilm formation and downstream virulence factors in PAO1.

Further, researchers have designed novel AI mimics that interact with the QS receptors and behave as antagonists, thereby repressing the QS‐regulated virulence genes. Small molecule inhibitors or antagonists have been developed to closely associate and target the different QS receptors of *P. aeruginosa*, thereby preventing signal reception (Suneby *et al*., 2017; Horita *et al*., 2018). Numerous antagonists have been shown to strongly interact and stabilize LasR, preventing its dimerization and making it incapable of binding DNA (Suneby *et al*., 2017). Similarly, antagonists targeting QscR (an orphan or LuxR ‘solo’ receptor) exhibited high specificity and successfully impeded the dimerization of QscR, abrogating its DNA binding activity (Horita *et al*., 2018). Derivatives of a small abiotic LasR antagonist, V‐06–018, demonstrated a 10‐ and 100‐fold greater potency than V‐06–018 and commonly used AHL‐based LasR antagonists, respectively, with higher specificity and selectivity for LasR by directly associating with the native ligand‐binding site in LasR and stabilizing this inactive form (Manson *et al*., 2020). Despite being low molecular weight and non‐proteinaceous in nature, AHLs (QSSMs) have been shown to elicit antibody‐based immune responses. This property has been exploited to generate monoclonal antibodies (mAbs) targeting AHLs for inactivation, sequestration or degradation (Kalia *et al*., 2019). The mAb RS2‐1G9 targeting 3‐oxo‐C12‐HSL exhibited QQ properties, reduced pyocyanin production in *P. aeruginosa* and protected murine macrophages from 3‐oxo‐C12‐HSL‐mediated apoptosis (Kaufmann *et al*., 2006). Also, screening studies by Marin *et al*. (2007) identified a mAb, XYD‐11G2, to effectively degrade 3‐oxo‐C12‐HSL and inhibit pyocyanin production in *P. aeruginosa*. Although the application of mAb in QQ is worthwhile, it warrants further investigations to assert their specificity and effectiveness *in vivo*. Another emerging strategy that has been widely investigated to silence QS in *P. aeruginosa* directly exploits microbial enzymes. These QQ enzymes, including lactonases, acylases and paraoxonase, catalyse the degradation of QSSMs by either opening their lactone ring or hydrolysing the amide linkage to yield fatty acids and homoserine lactone (Kalia *et al*., 2019). QQ acylases have been reported to degrade 3‐oxo‐C12‐HSL, preventing its accumulation (Sio *et al*., 2006), altering the biofilm structure and transcriptome of PAO1 (de Celis *et al*., 2021), and attenuating its virulence in *C. elegans* and a mouse model of pulmonary infection (Utari *et al*., 2018). Microbial lactonases have exhibited promising QQ potential by attenuating QS and virulence in clinical strains of *P. aeruginosa* isolated from burn wounds (López‐Jácome *et al*., 2019) and also reduced mortality in a murine model of pneumonia by ~fourfold, without altering the bacterial lung count (Hraiech *et al*., 2014). Paraoxonase isolated from mammalian cells has also been shown to inhibit AHL‐mediated QS in *P. aeruginosa* (Yang *et al*., 2005; Stoltz *et al*., 2007). These evidences build a strong foundation for exploiting alternative anti‐virulence drugs in combating pseudomonal infections.

## Bridging the gap between laboratory and market: data from clinical and field trials

The mammoth literature on the anti‐virulence prospects of bioactive phytochemicals is majorly collated from bench‐based experiments and investigations with animal models. However, there is a lack of scientific knowledge from clinical settings and field trials discussing their safety and possible toxic effects. In contrast, most of the field studies circumference around other types of QSI, including antibiotics and synthetic drugs. A pilot‐scale clinical trial was undertaken to evaluate the therapeutic efficacy of garlic oil macerate as a QSI against *P. aeruginosa* in 26 cystic fibrosis patients (Smyth *et al*., 2010). Garlic capsules at concentrations lower than the toxicity dose for humans (656 mg) were consumed daily for 8 weeks, and the clinical outcomes were measured at baseline and post‐treatment. Patients from the treatment group displayed improved pulmonary function with a significant reduction in disease symptoms. However, a small fraction of patients exhibited abnormal liver function and minor adverse effects (Smyth *et al*., 2010). In a controlled study, the anti‐QS potential of azithromycin was assessed in intubated patients colonized by *P. aeruginosa* (Köhler *et al*., 2010). Azithromycin was administered intravenously (300 mg per day) for 20 days, and tracheal aspirates were collected each day. Following treatment with azithromycin, the expression of QS genes (*lasI* and *rhlA*) in tracheal samples was significantly reduced. However, this treatment strategy remained dubious as it may drive microbial evolution leading to the selection of resistant strains that are unresponsive to the QSI. In a similar direction, a randomized controlled trial was on intubated patients vulnerable to ventilator‐associated pneumonia caused by *P. aeruginosa* (van Delden *et al*., 2012). *P. aeruginosa* was drastically reduced following treatment with azithromycin (300 mg per day for 20 days). The QS‐regulated virulence of colonizing *P. aeruginosa* isolates remained low, and azithromycin treatment resulted in a fivefold reduction in the incidence of high‐level rhamnolipids producing isolates among the high‐risk patient subgroup. Interestingly, Wahjudi and colleagues developed a novel inhalable system by freeze drying an AHL acylase (PvdQ) with anti‐QS capabilities to attenuate the virulence of colonizing *P. aeruginosa* in cystic fibrosis patients (Wahjudi *et al*., 2013). The formulation was found to be stable up to four weeks even at 55°C. However, it warrants clinical investigations to assess its therapeutic and safety index. Fimbrolide‐coated contact lenses were monitored for their clinical safety against multiple human pathogens, including *P. aeruginosa* in guinea pigs and human subjects, for 30 days and 24 h respectively (Zhu *et al*., 2008). The contact lenses exhibited QQ properties and showed that bacterial adherence was reduced by 67–92%, asserting its bioremedial ability. In clinical set‐ups, indwelling medical devices act as a focal point for initiating health care‐associated infections, particularly catheter‐associated urinary tract infections (Saini *et al*., 2017). Urinary catheters functionalized by coating with 5‐fluorouracil, a previously characterized QSI (Ueda *et al*., 2009), have demonstrated effective antimicrobial properties with no episodes of catheter‐related bacteraemia or any adverse effects in ICU patients (Walz *et al*., 2010). Cumulatively, these trials can be considered imperative in their attempts to translate bench‐based researches on QSI. Such investigations can encourage further trials that may deal with a wide range of QSI, specifically QQ phytochemicals, their potential toxicity and therapeutic applications in the medical sector. Collaborative efforts from the pharmaceutical industry and research‐directed academia can help bridge this gap for repurposing bioactive phytochemicals for anti‐virulence therapy. Until then, studies in this avenue are still in their infancy.

## Weighing the outcomes on scales: benefits and pitfalls of using QQ phytochemicals

The discovery of antibiotics was considered to be a pinnacle in the field of medicine. Unfortunately, indiscriminate use and unregulated consumption of these antimicrobial drugs have led to the emergence of MDR pathogens (Chadha *et al*., 2021c). Notably, the COVID‐19 pandemic and the emergence of SARS‐CoV‐2 variants (Chadha *et al*., 2021b) have increased the consumption of multiple antibiotics globally (Adebisi *et al*., 2021). This sudden ‘antibiotic rush’ can accelerate the pace at which bacterial resistance evolves and severely compromise the global commitment to curb AMR (Pelfrene *et al*., 2021). Despite the advent of new generation antibiotics, bacterial infections continue to rise, posing a continuous risk to the global healthcare system (Kalia *et al*., 2007). With the onset of the post‐antibiotic era, anti‐virulence drugs (QSIs) have caught the attention of the scientific community. These ‘wonder drugs’ include bioactive phytochemicals that interfere with the expression of virulence genes without killing the pathogenic bacteria or impeding their growth. Hence, this strategy exerts a relatively lower selection pressure as compared to antimicrobial drugs. Contrarily, since evolution is considered to be the only constant in the microbial world, it is believed that these QQ compounds may also impose selection pressure resulting in the development of drug resistance (Kalia *et al*., 2019). Although there is very little evidence to support this concern (Kalia *et al*., 2014; Koul *et al*., 2016), this line of thought seems critical as no treatment strategy be considered full‐proof. However, a combinational therapy employing QQ phytochemicals and antibiotics can increase the success rate of drug repurposing by targeting multiple bacterial signalling pathways, thereby reducing the selection pressure. In this regard, studies illustrating the synergistic effects of anti‐virulence phytochemicals and antibiotics have surfaced recently (Sharma *et al*., 2020a; Bose *et al*., 2021). Nonetheless, more insights are warranted in this avenue to substantiate the use of potent phytochemical–antibiotic regimens.

In addition, bacterial pathogens may reiterate their metabolic pathways or select mutations that confer resistance to QSIs. Clinical strains of *P. aeruginosa* isolated from cystic fibrosis patients were shown to harbour resistance against QSIs such as brominated furanone by inducing genomic alterations that enhance bacterial efflux capabilities (Maeda *et al*., 2012). Hence, bacterial pathogens can evolve artilleries that disrupt QQ by different mechanisms. Interestingly, reports have identified QS mutants of *P. aeruginosa* that are unresponsive to the QSSMs (Sandoz *et al*., 2007). These QS mutants are termed ‘social cheaters’ and display altered fitness with reduced chances of survival. However, such mutants can deceive QSIs, belittling their therapeutic value and lowering their efficacy (Gerdt and Blackwell, 2014). Therefore, like any other anti‐virulence drug, QQ phytochemicals can also share the same fate. Another dimension to pseudomonal virulence is the presence of three distinct but interconnected QS mechanisms, making it an uphill task for targeting by QQ phytochemicals. Moreover, the presence of multiple QS systems increases the possibility that heterodimers of transcriptional regulators can associate with different promoters indiscriminately, driving differential gene expression in bacteria as a countermeasure to evade QSIs (Koul and Kalia, 2017). Just like the pseudomonal Pqs system that regulates the expression of virulence factors that drive apoptosis, which in turn induces the release of eDNA, conferring survival advantages to the pathogen (Hazan *et al*., 2016). Bacterial pathogens also can modify their QS receptors for enhanced binding by substituting the amino acid residues of the QSSM‐binding pocket (Hawkins *et al*., 2007). Hence, QSIs exhibiting a relatively high affinity towards the QS receptors will be more effective in disarming the pathogen's virulence. This may prove to be a setback when employing natural anti‐QS phytochemicals but advantageous with chemically modified ones with desired functional groups that facilitate stronger interactions.

On the positive front, bioactive phytochemicals play an imperial role in modulating the host immune system by activating humoral and cell‐mediated responses, supplying antioxidants, impeding inflammatory responses and autoimmune disorders, and maintaining a healthy gut microbiome (Yin *et al*., 2019; Behl *et al*., 2021). Although they offer endless benefits, their role in modulating the diversity, abundance and equilibrium of the human microbiome has not been well documented. Considering the far‐reaching effects of this versatile organ (human microbiome) in various diseases, including cancers, its role in shaping human well‐being cannot be undermined (Chadha *et al*., 2021d). Therefore, QQ phytochemicals can be anticipated to impact human health by modulating the microbiome of different organs of the body. Other major limitations of using phytochemicals in anti‐virulence therapy are their cytotoxic effects, poor solubility in water and inadequate bioavailability. Nevertheless, these can be regulated for human benefit by fabricating suitable delivery systems for achieving a high therapeutic index. Therefore, the desirable properties of an ideal QQ phytochemical can be enlisted as follows: (i) low molecular weight; (ii) high specificity towards QS machinery with no toxic or off‐target effects in the bacterial pathogen; (iii) high stability and adequate bioavailability; (iv) resistant to enzymatic degradation within the host system; (v) minimal or no cytotoxicity for the host, i.e. safe for human use; and (vi) no exertion of harmful effects over the host microbiome. Nevertheless, such naturopathic QSIs warrant more scientific explorations in clinical studies and their ability to withstand the sands of time by overcoming the risk of antimicrobial resistance. It will then be a matter of time before such potent anti‐virulence drugs enter into clinical practices.

## Conclusion and prospective outlook

With the indiscriminate use of antibiotics in clinical settings, *P. aeruginosa* has emerged as a highly notorious and multi‐drug‐resistant pathogen posing a serious threat to public healthcare systems. Since antimicrobial therapies work by inhibiting bacterial growth or killing them, they continue to pose an intense selection pressure favouring the selection of resistant variants in the population. The spurring fidelity between ever‐evolving AMR and advancements in antimicrobial therapies has rattled clinicians worldwide to seek alternative medicine that attenuates pathogen virulence without compromising microbial growth. Also, considering the slow pace at which newer drugs get the safety and administrative approvals, drugs that exhibit both antimicrobial and anti‐virulent traits may serve as an ideal intervention strategy for future action. The ‘disarm‐don’t kill’ anti‐virulence approach can also be revitalized to develop effective, robust, pathogen‐specific drugs. Therefore, targeting the QS machinery that supervises virulence in *P. aeruginosa* can be vital to limiting the severity of infections in clinical set‐ups. In this context, bioactive phytochemicals from plant varieties have come to the rescue. These natural anti‐virulence elixirs have unravelled newer prospects for researchers to explore and develop novel therapeutics against *P. aeruginosa* that disarm its QS mechanisms, attenuating its virulence determinants. The last two decades have witnessed unprecedented research on this aspect and indicated the QQ prospects of numerous plant bioactives. Secondary metabolites that influence the pathogenesis of *P. aeruginosa* with anti‐QS and anti‐biofilm potential have also been recognized from bench‐based studies *in vitro* and experiments with animal models *in vivo*. However, these require further validation for their successful transition and translation into applied medicine for combating pseudomonal infections in clinical set‐ups. Rather than designing new antibiotics, scientific experimentations should be directed to identify or repurpose natural products, bioactive compounds, synthetic drugs, *etc*. with anti‐virulent properties and the development of compatible nanocarriers for better delivery and bioavailability. These compounds may be subjected to chemical or structural modification(s) for improved binding to putative drug targets of the bacterial QS systems. Moreover, greater emphasis must be laid to boost large‐scale clinical trials on the effects of QSIs on various infectious diseases. This will generate concrete and reliable data before this anti‐virulence strategy can be exploited for the benefit of mankind. It can be said that the mammoth scientific journey for these novel bioactives has crossed its halfway mark. With the advent of advanced molecular techniques, the age of molecular medicine is leading to the revival and modernization of Ayurvedic medicine. This has opened another vast avenue augmenting plant bioactives and nanotechnology for enhanced and targeted drug delivery. The future outlook may include translational approaches such as the ‘Trojan Horse’ strategy for developing novel conjugates of QQ phytochemicals and bacterial virulence factors for targeted and improved delivery. This can prove to be path‐breaking in circumventing the menace of AMR. Also, it is vital to study the prevalence of QSSM‐unresponsive mutants of *P. aeruginosa* using large‐scale screening studies. By estimating the clinical prevalence of such QS mutants, a dual drug regimen harbouring both antimicrobial and anti‐virulence properties may be employed to counter these cell populations. Presently, there is not enough evidence to support the development of QSI‐induced resistance in pathogenic microorganisms. Since the mechanism of the QSI also influences the possibility of developing resistance to QSI, it becomes essential to discretely investigate how anti‐virulence drugs can induce anthropogenic resistance in pathogenic bacteria. Since pseudomonal biofilms on medical devices are the grim instigator in most nosocomial infections, fabricating multilayer coatings of bioactive phytochemicals and their nanoformulations can boost the biocurative potential of such nanoscale materials. Also, hybrid nanocoatings of multiple phytochemicals and/or QQ enzymes such as acylases, paraoxonase and lactonases on indwelling medical devices can be developed for imparting anti‐biofilm activities holistically. These advancements can aid in reducing the global reliance on antibiotics. Therefore, dedicated investigations from academia and attention from the pharmaceutical industries can boost the innovation and recognition of novel plant‐derived anti‐pseudomonal drugs. Overall, enacting such initiatives can help reduce the incidence of drug resistance and the global medical burden imposed by *P. aeruginosa*, thereby reducing hospital stays and improving the quality of patient life. This will also provide substantial societal and economic returns, essential to improving lives today and for future generations.

## Conflict of interest

None declared.
